# Supplementary material for: Artificial intelligence-based modeling for accurate leaf area estimation in olive (Olea europaea L.) cultivars
Source: PLoS One. 2026 Jan 2;21(1):e0339865. doi: 10.1371/journal.pone.0339865 (PMC12758791; doi:10.1371/journal.pone.0339865)
Supplement: S1 Table — (DOCX) [file pone.0339865.s001.docx]

**S1 Table.** Descriptive statistics for individual olive cultivars used in this study.

| Cultivar  name | Number of cultivars |  | Training (70%) | | | Testing (30%) | | | All (100%) | | |
| --- | --- | --- | --- | --- | --- | --- | --- | --- | --- | --- | --- |
|  |  |  | LL (mm) | LW (mm) | LA (cm^2^) | LL (mm) | LW (mm) | LA (cm^2^) | LL (mm) | LW (mm) | LA (cm^2^) |
|  |  | Min. | 28.13 | 7.70 | 1.75 | 16.59 | 6.61 | 0.86 | 16.59 | 6.61 | 0.86 |
|  |  | Max. | 55.00 | 22.52 | 8.68 | 40.32 | 10.61 | 2.98 | 55.00 | 22.52 | 8.68 |
|  |  | Avg. | 40.00 | 12.64 | 3.95 | 25.44 | 8.09 | 1.54 | 35.39 | 11.20 | 3.19 |
| Arbequina' | 1 | SE | 1.10 | 0.68 | 0.32 | 1.70 | 0.28 | 0.15 | 1.27 | 0.55 | 0.27 |
|  |  | SD | 7.03 | 4.38 | 2.08 | 7.39 | 1.21 | 0.64 | 9.75 | 4.21 | 2.06 |
|  |  | Cv | 0.18 | 0.35 | 0.53 | 0.29 | 0.15 | 0.42 | 0.28 | 0.38 | 0.65 |
|  |  | Skew | -0.70 | 0.01 | 0.53 | -0.21 | -0.43 | 0.67 | -0.13 | 1.37 | 1.40 |
|  |  | Kurt | 0.29 | 1.09 | 1.29 | 0.83 | 0.84 | 1.16 | -0.72 | 0.93 | 1.25 |
|  |  | Min. | 29.27 | 7.97 | 1.80 | 25.30 | 7.54 | 1.43 | 25.30 | 7.54 | 1.43 |
|  |  | Max. | 88.23 | 18.86 | 10.49 | 55.53 | 13.03 | 5.65 | 88.23 | 18.86 | 10.49 |
|  |  | Avg. | 57.75 | 12.45 | 5.63 | 41.89 | 9.48 | 3.09 | 52.73 | 11.51 | 4.83 |
| Ayvalık' | 2 | SE | 2.20 | 0.47 | 0.38 | 1.68 | 0.31 | 0.22 | 1.85 | 0.38 | 0.31 |
|  |  | SD | 14.10 | 3.01 | 2.45 | 7.33 | 1.35 | 0.94 | 14.25 | 2.91 | 2.38 |
|  |  | Cv | 0.24 | 0.24 | 0.43 | 0.18 | 0.14 | 0.30 | 0.27 | 0.25 | 0.49 |
|  |  | Skew | -0.85 | -0.95 | -1.23 | 0.66 | 1.27 | 1.99 | 0.55 | 0.82 | 0.85 |
|  |  | Kurt | 0.26 | 0.47 | 0.45 | -0.12 | 0.81 | 1.00 | -0.51 | -0.38 | -0.57 |
|  |  | Min. | 27.52 | 8.29 | 1.94 | 32.22 | 7.67 | 2.17 | 27.52 | 7.67 | 1.94 |
|  |  | Max. | 74.72 | 22.02 | 10.18 | 54.27 | 16.29 | 5.46 | 74.72 | 22.02 | 10.18 |
|  |  | Avg. | 55.29 | 15.15 | 5.95 | 42.37 | 12.87 | 3.81 | 51.20 | 14.42 | 5.28 |
| Çelebi' | 3 | SE | 1.92 | 0.51 | 0.32 | 1.61 | 0.53 | 0.19 | 1.60 | 0.41 | 0.26 |
|  |  | SD | 12.30 | 3.27 | 2.07 | 7.02 | 2.31 | 0.82 | 12.32 | 3.14 | 2.02 |
|  |  | Cv | 0.22 | 0.22 | 0.35 | 0.17 | 0.18 | 0.22 | 0.24 | 0.22 | 0.38 |
|  |  | Skew | -0.71 | 0.09 | -0.39 | -1.16 | -0.08 | 0.44 | 0.16 | 0.34 | 0.47 |
|  |  | Kurt | -0.35 | 0.23 | -0.06 | 0.37 | -0.49 | 0.43 | -1.02 | 0.22 | -0.49 |
|  |  | Min. | 23.36 | 7.96 | 1.74 | 19.77 | 6.07 | 1.07 | 19.77 | 6.07 | 1.07 |
|  |  | Max. | 76.34 | 22.04 | 14.58 | 39.89 | 11.82 | 4.13 | 76.34 | 22.04 | 14.58 |
|  |  | Avg. | 50.73 | 13.92 | 6.60 | 30.01 | 8.14 | 2.24 | 44.52 | 12.18 | 5.30 |
| Domat' | 4 | SE | 2.18 | 0.61 | 0.55 | 1.34 | 0.38 | 0.19 | 2.00 | 0.56 | 0.47 |
|  |  | SD | 14.13 | 3.98 | 3.60 | 5.69 | 1.62 | 0.80 | 15.36 | 4.31 | 3.61 |
|  |  | Cv | 0.28 | 0.29 | 0.54 | 0.19 | 0.20 | 0.36 | 0.34 | 0.35 | 0.68 |
|  |  | Skew | -0.67 | -0.81 | -0.55 | -0.94 | 0.29 | 0.55 | 0.61 | 0.63 | 1.09 |
|  |  | Kurt | 0.44 | 0.44 | 0.83 | -0.12 | 1.03 | 0.78 | -0.50 | -0.61 | 0.09 |
|  |  | Min. | 27.99 | 4.53 | 1.46 | 24.52 | 5.95 | 2.00 | 24.52 | 4.53 | 1.46 |
|  |  | Max. | 87.52 | 16.86 | 9.76 | 60.18 | 15.26 | 6.28 | 87.52 | 16.86 | 9.76 |
|  |  | Avg. | 56.45 | 10.62 | 5.35 | 39.86 | 10.55 | 3.44 | 51.10 | 10.60 | 4.74 |
| Edincik Su' | 5 | SE | 2.43 | 0.49 | 0.42 | 2.06 | 0.69 | 0.27 | 2.04 | 0.39 | 0.32 |
|  |  | SD | 15.54 | 3.12 | 2.66 | 8.74 | 2.92 | 1.13 | 15.65 | 2.98 | 2.43 |
|  |  | Cv | 0.28 | 0.29 | 0.50 | 0.22 | 0.28 | 0.33 | 0.31 | 0.28 | 0.51 |
|  |  | Skew | -1.10 | -0.72 | -1.37 | 0.33 | -1.08 | 0.66 | 0.51 | -0.01 | 0.76 |
|  |  | Kurt | 0.17 | 0.01 | 0.32 | 0.55 | -0.10 | 0.91 | -0.81 | -0.81 | -0.75 |
|  |  | Min. | 13.39 | 5.49 | 0.60 | 12.52 | 5.31 | 0.56 | 12.52 | 5.31 | 0.56 |
|  |  | Max. | 44.40 | 13.38 | 3.82 | 23.83 | 9.74 | 1.76 | 44.40 | 13.38 | 3.82 |
|  |  | Avg. | 29.10 | 9.71 | 2.26 | 19.39 | 6.83 | 1.05 | 26.19 | 8.85 | 1.90 |
| Elmacık' | 6 | SE | 1.09 | 0.30 | 0.12 | 0.67 | 0.27 | 0.07 | 0.98 | 0.28 | 0.11 |
|  |  | SD | 7.09 | 1.95 | 0.75 | 2.84 | 1.15 | 0.28 | 7.51 | 2.17 | 0.85 |
|  |  | Cv | 0.24 | 0.20 | 0.33 | 0.15 | 0.17 | 0.27 | 0.29 | 0.25 | 0.45 |
|  |  | Skew | -0.26 | -0.16 | -0.10 | 0.50 | 0.79 | 1.12 | 0.43 | 0.07 | 0.22 |
|  |  | Kurt | 0.03 | -0.43 | -0.31 | -0.56 | 0.87 | 0.72 | -0.57 | -1.02 | -0.99 |
|  |  | Min. | 36.15 | 11.03 | 3.02 | 36.40 | 9.66 | 2.54 | 36.15 | 9.66 | 2.54 |
|  |  | Max. | 75.03 | 29.78 | 14.17 | 68.94 | 20.92 | 9.12 | 75.03 | 29.78 | 14.17 |
|  |  | Avg. | 61.27 | 20.09 | 8.89 | 49.29 | 13.81 | 4.93 | 57.67 | 18.21 | 7.70 |
| Frantoio' | 7 | SE | 1.65 | 0.76 | 0.45 | 2.18 | 0.60 | 0.42 | 1.50 | 0.68 | 0.41 |
|  |  | SD | 10.71 | 4.96 | 2.89 | 9.24 | 2.56 | 1.79 | 11.52 | 5.19 | 3.14 |
|  |  | Cv | 0.17 | 0.25 | 0.33 | 0.19 | 0.19 | 0.36 | 0.20 | 0.28 | 0.41 |
|  |  | Skew | -0.20 | -0.83 | -0.52 | -0.50 | 2.63 | 1.08 | -0.29 | 0.52 | 0.16 |
|  |  | Kurt | -0.83 | 0.23 | -0.27 | 0.63 | 1.14 | 1.13 | -1.17 | -0.72 | -1.04 |
|  |  | Min. | 26.42 | 6.03 | 1.43 | 50.34 | 13.54 | 3.73 | 22.91 | 6.03 | 1.10 |
|  |  | Max. | 84.84 | 16.75 | 9.51 | 22.91 | 6.20 | 1.10 | 84.84 | 16.75 | 9.51 |
|  |  | Avg. | 53.34 | 12.87 | 5.26 | 34.86 | 9.61 | 2.50 | 47.80 | 11.89 | 4.43 |
| Gemlik' | 8 | SE | 2.11 | 0.42 | 0.33 | 1.77 | 0.51 | 0.19 | 1.91 | 0.38 | 0.29 |
|  |  | SD | 13.70 | 2.73 | 2.14 | 7.50 | 2.16 | 0.81 | 14.70 | 2.94 | 2.22 |
|  |  | Cv | 0.26 | 0.21 | 0.41 | 0.22 | 0.22 | 0.33 | 0.31 | 0.25 | 0.50 |
|  |  | Skew | -0.52 | 0.14 | -0.87 | 0.24 | -0.74 | -0.80 | 0.41 | -0.21 | 0.52 |
|  |  | Kurt | 0.17 | -0.68 | 0.11 | 0.39 | 0.26 | 0.22 | -0.60 | -0.85 | -0.80 |
|  |  | Min. | 24.09 | 6.31 | 1.44 | 33.04 | 7.45 | 2.22 | 24.09 | 6.31 | 1.44 |
|  |  | Max. | 81.47 | 20.84 | 9.31 | 62.23 | 13.46 | 6.19 | 81.47 | 20.84 | 9.31 |
|  |  | Avg. | 54.67 | 13.16 | 5.62 | 48.65 | 11.03 | 4.14 | 52.86 | 12.52 | 5.17 |
| Gemlik-21' | 9 | SE | 2.15 | 0.56 | 0.37 | 2.01 | 0.37 | 0.28 | 1.65 | 0.42 | 0.28 |
|  |  | SD | 13.92 | 3.60 | 2.39 | 8.55 | 1.58 | 1.18 | 12.68 | 3.24 | 2.18 |
|  |  | Cv | 0.25 | 0.27 | 0.43 | 0.18 | 0.14 | 0.29 | 0.24 | 0.26 | 0.42 |
|  |  | Skew | -0.50 | -0.40 | -1.06 | -0.71 | 0.08 | -0.75 | -0.19 | 0.49 | 0.13 |
|  |  | Kurt | -0.48 | 0.14 | -0.28 | 0.10 | -0.63 | 0.31 | -0.59 | 0.01 | -0.99 |
|  |  | Min. | 19.97 | 5.31 | 0.79 | 21.03 | 6.42 | 1.40 | 19.97 | 5.31 | 0.79 |
|  |  | Max. | 42.85 | 14.38 | 4.05 | 56.46 | 15.40 | 6.02 | 56.46 | 15.40 | 6.02 |
|  |  | Avg. | 30.32 | 10.11 | 2.25 | 32.91 | 11.20 | 2.74 | 31.09 | 10.44 | 2.40 |
| Girit Zeytini' | 10 | SE | 0.96 | 0.39 | 0.12 | 2.48 | 0.68 | 0.33 | 1.00 | 0.35 | 0.13 |
|  |  | SD | 6.25 | 2.55 | 0.79 | 10.50 | 2.88 | 1.40 | 7.70 | 2.65 | 1.02 |
|  |  | Cv | 0.21 | 0.25 | 0.35 | 0.32 | 0.26 | 0.51 | 0.25 | 0.25 | 0.42 |
|  |  | Skew | -0.80 | -0.99 | -0.18 | 0.27 | -0.95 | 0.21 | 0.95 | -0.10 | 1.19 |
|  |  | Kurt | 0.19 | -0.25 | 0.40 | 1.02 | -0.06 | 1.06 | 1.07 | -0.86 | 1.69 |
|  |  | Min. | 27.33 | 10.31 | 2.46 | 17.49 | 5.67 | 1.11 | 17.49 | 5.67 | 1.11 |
|  |  | Max. | 62.40 | 20.15 | 9.44 | 31.51 | 10.86 | 2.41 | 62.40 | 20.15 | 9.44 |
|  |  | Avg. | 41.15 | 13.72 | 4.32 | 24.08 | 8.81 | 1.65 | 36.03 | 12.25 | 3.52 |
| Halhalı' | 11 | SE | 1.49 | 0.34 | 0.24 | 1.09 | 0.36 | 0.10 | 1.49 | 0.39 | 0.23 |
|  |  | SD | 9.67 | 2.22 | 1.52 | 4.63 | 1.52 | 0.44 | 11.45 | 3.01 | 1.77 |
|  |  | Cv | 0.23 | 0.16 | 0.35 | 0.19 | 0.17 | 0.26 | 0.32 | 0.25 | 0.50 |
|  |  | Skew | -1.12 | 0.53 | 1.61 | -1.37 | -0.78 | -0.99 | 0.27 | 0.14 | 0.85 |
|  |  | Kurt | 0.10 | 0.83 | 1.20 | -0.02 | -0.21 | 0.60 | -1.01 | -0.22 | 0.60 |
|  |  | Min. | 24.12 | 8.19 | 1.89 | 22.90 | 6.20 | 1.20 | 22.90 | 6.20 | 1.20 |
|  |  | Max. | 57.90 | 27.23 | 10.18 | 50.90 | 12.27 | 3.43 | 57.90 | 27.23 | 10.18 |
|  |  | Avg. | 45.82 | 15.23 | 5.02 | 33.49 | 8.64 | 1.98 | 42.12 | 13.25 | 4.11 |
| Karamani' | 12 | SE | 1.35 | 0.70 | 0.31 | 1.83 | 0.39 | 0.14 | 1.31 | 0.64 | 0.29 |
|  |  | SD | 8.73 | 4.55 | 2.02 | 7.76 | 1.67 | 0.59 | 10.05 | 4.90 | 2.20 |
|  |  | Cv | 0.19 | 0.30 | 0.40 | 0.23 | 0.19 | 0.30 | 0.24 | 0.37 | 0.54 |
|  |  | Skew | -0.24 | -0.19 | -0.04 | -0.09 | -0.29 | 1.46 | -0.18 | 0.62 | 0.69 |
|  |  | Kurt | -0.51 | 0.38 | 0.47 | 0.52 | 0.44 | 1.16 | -0.93 | -0.36 | -0.27 |
|  |  | Min. | 23.01 | 7.99 | 1.74 | 23.25 | 6.70 | 1.26 | 23.01 | 6.70 | 1.26 |
|  |  | Max. | 93.81 | 17.41 | 11.08 | 45.39 | 13.42 | 3.41 | 93.81 | 17.41 | 11.08 |
|  |  | Avg. | 58.61 | 14.11 | 6.05 | 32.13 | 10.53 | 2.40 | 50.67 | 13.04 | 4.95 |
| Kilis Yağlık' | 13 | SE | 3.38 | 0.37 | 0.43 | 1.74 | 0.37 | 0.13 | 2.88 | 0.35 | 0.37 |
|  |  | SD | 21.92 | 2.42 | 2.81 | 7.39 | 1.58 | 0.54 | 22.16 | 2.72 | 2.88 |
|  |  | Cv | 0.37 | 0.17 | 0.46 | 0.23 | 0.15 | 0.23 | 0.44 | 0.21 | 0.58 |
|  |  | Skew | -1.42 | 0.04 | -1.17 | -1.17 | 0.78 | 0.04 | 0.74 | -0.25 | 0.75 |
|  |  | Kurt | 0.37 | -0.89 | 0.34 | 0.54 | -0.47 | -0.13 | -0.84 | -1.03 | -0.76 |
|  |  | Min. | 33.44 | 9.38 | 2.79 | 32.01 | 5.80 | 1.36 | 32.01 | 5.80 | 1.36 |
|  |  | Max. | 85.43 | 20.99 | 10.36 | 47.60 | 14.32 | 3.63 | 85.43 | 20.99 | 10.36 |
|  |  | Avg. | 61.37 | 14.77 | 6.66 | 38.95 | 9.74 | 2.73 | 54.64 | 13.26 | 5.48 |
| Manzanilla' | 14 | SE | 2.35 | 0.35 | 0.34 | 0.97 | 0.57 | 0.16 | 2.13 | 0.42 | 0.34 |
|  |  | SD | 15.22 | 2.27 | 2.22 | 4.09 | 2.42 | 0.67 | 16.39 | 3.24 | 2.59 |
|  |  | Cv | 0.25 | 0.15 | 0.33 | 0.11 | 0.25 | 0.24 | 0.30 | 0.24 | 0.47 |
|  |  | Skew | -1.49 | 0.53 | -1.23 | -0.43 | -0.83 | -0.78 | 0.47 | -0.33 | 0.27 |
|  |  | Kurt | 0.01 | 0.08 | -0.15 | 0.34 | 0.17 | -0.50 | -1.26 | -0.33 | -1.31 |
|  |  | Min. | 29.80 | 7.53 | 1.70 | 26.07 | 6.46 | 1.46 | 26.07 | 6.46 | 1.46 |
|  |  | Max. | 74.64 | 19.01 | 8.35 | 47.47 | 12.96 | 4.45 | 74.64 | 19.01 | 8.35 |
|  |  | Avg. | 50.57 | 13.11 | 5.00 | 36.06 | 10.01 | 2.75 | 46.22 | 12.18 | 4.33 |
| Memecik' | 15 | SE | 1.86 | 0.34 | 0.27 | 1.49 | 0.47 | 0.21 | 1.62 | 0.33 | 0.24 |
|  |  | SD | 12.04 | 2.22 | 1.75 | 6.31 | 1.99 | 0.90 | 12.43 | 2.55 | 1.84 |
|  |  | Cv | 0.24 | 0.17 | 0.35 | 0.17 | 0.20 | 0.33 | 0.27 | 0.21 | 0.43 |
|  |  | Skew | -0.90 | 1.33 | -0.87 | -0.55 | -1.20 | -0.75 | 0.35 | -0.21 | 0.35 |
|  |  | Kurt | -0.04 | -0.39 | -0.02 | 0.22 | -0.19 | 0.51 | -0.89 | -0.12 | -0.93 |
|  |  | Min. | 30.77 | 10.28 | 2.35 | 26.07 | 6.46 | 1.46 | 26.49 | 7.89 | 1.51 |
|  |  | Max. | 76.43 | 21.64 | 11.08 | 47.47 | 12.96 | 4.45 | 76.43 | 21.64 | 11.08 |
|  |  | Avg. | 47.78 | 16.01 | 5.41 | 34.87 | 11.70 | 3.00 | 43.91 | 14.72 | 4.69 |
| Nizip Yağlık' | 16 | SE | 1.68 | 0.42 | 0.29 | 1.44 | 0.58 | 0.23 | 1.47 | 0.43 | 0.26 |
|  |  | SD | 10.92 | 2.73 | 1.87 | 6.10 | 2.48 | 0.96 | 11.27 | 3.27 | 1.97 |
|  |  | Cv | 0.23 | 0.17 | 0.35 | 0.17 | 0.21 | 0.32 | 0.26 | 0.22 | 0.42 |
|  |  | Skew | 1.12 | -0.36 | 2.56 | -0.95 | 0.44 | -0.24 | 0.92 | -0.10 | 1.11 |
|  |  | Kurt | 0.97 | -0.24 | 1.32 | 0.23 | 0.67 | 0.62 | 0.98 | -0.83 | 1.83 |
|  |  | Min. | 17.48 | 6.90 | 1.04 | 26.49 | 7.89 | 1.51 | 16.05 | 6.26 | 1.04 |
|  |  | Max. | 73.34 | 18.78 | 10.76 | 46.03 | 17.54 | 4.96 | 73.34 | 18.78 | 10.76 |
|  |  | Avg. | 38.94 | 11.27 | 3.69 | 20.98 | 8.88 | 1.49 | 33.55 | 10.55 | 3.03 |
| Sarı Haşebi' | 17 | SE | 2.67 | 0.40 | 0.38 | 0.72 | 0.40 | 0.07 | 2.16 | 0.33 | 0.30 |
|  |  | SD | 17.29 | 2.56 | 2.48 | 3.05 | 1.69 | 0.30 | 16.57 | 2.55 | 2.29 |
|  |  | Cv | 0.44 | 0.23 | 0.67 | 0.15 | 0.19 | 0.20 | 0.49 | 0.24 | 0.75 |
|  |  | Skew | -0.59 | 1.41 | 0.81 | -1.26 | 1.26 | -0.62 | 1.19 | 1.13 | 1.68 |
|  |  | Kurt | 0.80 | 1.18 | 1.30 | -0.39 | 1.07 | 0.76 | 0.23 | 1.37 | 2.00 |
|  |  | Min. | 34.50 | 7.92 | 1.99 | 22.77 | 7.07 | 1.46 | 22.77 | 7.07 | 1.46 |
|  |  | Max. | 74.73 | 21.88 | 11.40 | 25.05 | 12.66 | 2.08 | 74.73 | 21.88 | 11.40 |
|  |  | Avg. | 52.66 | 16.01 | 6.04 | 37.88 | 11.06 | 3.02 | 48.23 | 14.53 | 5.13 |
| Sarı Ulak' | 18 | SE | 1.43 | 0.51 | 0.30 | 2.26 | 0.77 | 0.33 | 1.49 | 0.52 | 0.29 |
|  |  | SD | 9.29 | 3.33 | 1.97 | 9.60 | 3.27 | 1.41 | 11.44 | 3.97 | 2.26 |
|  |  | Cv | 0.18 | 0.21 | 0.33 | 0.25 | 0.30 | 0.47 | 0.24 | 0.27 | 0.44 |
|  |  | Skew | -0.24 | -0.37 | 0.18 | -1.42 | 1.09 | -0.32 | -0.21 | 0.03 | 0.39 |
|  |  | Kurt | 0.10 | -0.07 | 0.53 | 0.24 | 0.38 | 0.92 | -0.19 | -0.94 | -0.25 |
|  |  | Min. | 38.49 | 11.75 | 3.65 | 23.33 | 7.47 | 1.49 | 23.33 | 7.47 | 1.49 |
|  |  | Max. | 87.01 | 22.14 | 14.13 | 49.75 | 13.74 | 5.04 | 87.01 | 22.14 | 14.13 |
|  |  | Avg. | 64.74 | 16.61 | 8.03 | 36.45 | 10.18 | 2.85 | 56.26 | 14.68 | 6.47 |
| Sarı Yaprak' | 19 | SE | 1.98 | 0.46 | 0.38 | 1.69 | 0.49 | 0.24 | 2.24 | 0.52 | 0.41 |
|  |  | SD | 12.82 | 2.98 | 2.46 | 7.17 | 2.06 | 1.00 | 17.17 | 3.99 | 3.17 |
|  |  | Cv | 0.20 | 0.18 | 0.31 | 0.20 | 0.20 | 0.35 | 0.31 | 0.27 | 0.49 |
|  |  | Skew | -1.19 | -0.93 | -0.17 | 0.05 | -1.05 | 0.47 | 0.02 | 0.06 | 0.25 |
|  |  | Kurt | -0.04 | 0.33 | 0.29 | 0.24 | 0.38 | 0.92 | -1.10 | -0.78 | -0.80 |
|  |  | Min. | 12.02 | 6.39 | 0.56 | 13.33 | 5.51 | 0.64 | 12.02 | 5.51 | 0.56 |
|  |  | Max. | 49.75 | 15.91 | 5.30 | 26.05 | 11.74 | 1.91 | 49.75 | 15.91 | 5.30 |
|  |  | Avg. | 32.30 | 10.58 | 2.74 | 18.49 | 7.29 | 1.09 | 28.16 | 9.59 | 2.24 |
| Saurani' | 20 | SE | 1.65 | 0.42 | 0.21 | 1.05 | 0.39 | 0.11 | 1.45 | 0.37 | 0.18 |
|  |  | SD | 10.66 | 2.73 | 1.36 | 4.44 | 1.65 | 0.46 | 11.10 | 2.86 | 1.38 |
|  |  | Cv | 0.33 | 0.26 | 0.50 | 0.24 | 0.23 | 0.42 | 0.39 | 0.30 | 0.61 |
|  |  | Skew | -0.94 | -0.94 | -0.92 | -0.73 | 1.65 | -1.09 | 0.20 | 0.36 | 0.53 |
|  |  | Kurt | -0.41 | 0.01 | 0.05 | 0.79 | 1.37 | 0.77 | -1.31 | -1.00 | -0.86 |
|  |  | Min. | 20.28 | 7.95 | 1.30 | 21.37 | 6.36 | 1.36 | 20.28 | 6.36 | 1.30 |
|  |  | Max. | 77.62 | 26.50 | 12.72 | 46.66 | 27.37 | 8.96 | 77.62 | 27.37 | 12.72 |
|  |  | Avg. | 53.31 | 15.89 | 6.47 | 37.95 | 15.19 | 4.55 | 48.70 | 15.68 | 5.89 |
| Tavşan Yüreği' | 21 | SE | 2.09 | 0.81 | 0.42 | 1.75 | 1.49 | 0.58 | 1.80 | 0.72 | 0.36 |
|  |  | SD | 13.56 | 5.24 | 2.73 | 7.43 | 6.34 | 2.46 | 13.81 | 5.50 | 2.75 |
|  |  | Cv | 0.25 | 0.33 | 0.42 | 0.20 | 0.42 | 0.54 | 0.28 | 0.35 | 0.47 |
|  |  | Skew | -0.67 | -0.91 | -0.77 | -0.22 | -0.95 | -1.05 | 0.35 | 0.09 | 0.20 |
|  |  | Kurt | 0.05 | -0.03 | 0.09 | -0.77 | 0.35 | 0.46 | -0.54 | -0.97 | -0.87 |
|  |  | Min. | 26.87 | 7.76 | 1.67 | 24.93 | 6.18 | 1.16 | 24.93 | 6.18 | 1.16 |
|  |  | Max. | 75.01 | 22.08 | 10.54 | 50.36 | 13.60 | 4.03 | 75.01 | 22.08 | 10.54 |
|  |  | Avg. | 51.25 | 13.86 | 5.47 | 38.00 | 10.36 | 2.86 | 47.27 | 12.81 | 4.69 |
| Uslu' | 22 | SE | 1.92 | 0.60 | 0.38 | 1.51 | 0.41 | 0.17 | 1.62 | 0.48 | 0.31 |
|  |  | SD | 12.47 | 3.87 | 2.43 | 6.41 | 1.75 | 0.72 | 12.44 | 3.70 | 2.37 |
|  |  | Cv | 0.24 | 0.28 | 0.44 | 0.17 | 0.17 | 0.25 | 0.26 | 0.29 | 0.51 |
|  |  | Skew | -0.73 | -0.52 | -1.01 | 0.62 | 1.68 | 0.68 | 0.26 | 0.73 | 0.60 |
|  |  | Kurt | -0.19 | 0.42 | 0.09 | -0.08 | -0.79 | -0.75 | -0.83 | -0.02 | -0.76 |

*Min* minimum, *Max* maximum, *Avg* average, *SE* standard error, *SD* standard deviation, *Cv* coefficient of variation, *Skew* Skewness, *Kurt* Kurtosis, *LA* leaf area, *LW* leaf width, *LL* leaf length
