# Supplementary material for: Artificial intelligence-based modeling for accurate leaf area estimation in olive (Olea europaea L.) cultivars
Source: PLoS One. 2026 Jan 2;21(1):e0339865. doi: 10.1371/journal.pone.0339865 (PMC12758791; doi:10.1371/journal.pone.0339865)
Supplement: S2 Table — (DOCX) [file pone.0339865.s002.docx]

**S2 Table.** Dummy (one-hot) coefficients for cultivars (reference: ‘Gemlik’) in the multiple linear regression model.

| Cultivars | CN | Coef | std_err | *p*value |
| --- | --- | --- | --- | --- |
| 1 | ‘Arbequina’ | 0.202 | 0.082 | 0.014 |
| 2 | ‘Ayvalık’ | 0.047 | 0.080 | 0.562 |
| 3 | ‘Çelebi’ | -0.365 | 0.080 | 0.000 |
| 4 | ‘Domat’ | 1.096 | 0.080 | 0.000 |
| 5 | ‘Edincik Su’ | 0.444 | 0.080 | 0.000 |
| 6 | ‘Elmacık’ | 0.407 | 0.090 | 0.000 |
| 7 | ‘Frantoio’ | 0.074 | 0.083 | 0.370 |
| 9 | ‘Gemlik-21’ | 0.025 | 0.080 | 0.749 |
| 10 | ‘Girit Zeytini’ | 0.162 | 0.082 | 0.048 |
| 11 | ‘Halhalı’ | 0.152 | 0.081 | 0.062 |
| 12 | ‘Karamani’ | -0.194 | 0.080 | 0.016 |
| 13 | ‘Kilis Yağlık’ | -0.154 | 0.080 | 0.054 |
| 14 | ‘Manzanilla’ | -0.110 | 0.080 | 0.168 |
| 15 | ‘Memecik’ | -0.022 | 0.080 | 0.786 |
| 16 | ‘Nizip Yağlık’ | -0.316 | 0.081 | 0.000 |
| 17 | ‘Sarı Haşebi’ | 0.415 | 0.081 | 0.000 |
| 18 | ‘Sarı Ulak’ | -0.245 | 0.080 | 0.002 |
| 19 | ‘Sarı Yaprak’ | 0.227 | 0.080 | 0.005 |
| 20 | ‘Saurani’ | 0.172 | 0.090 | 0.058 |
| 21 | ‘Tavşan Yüreği’ | 0.054 | 0.081 | 0.507 |
| 22 | ‘Uslu’ | 0.014 | 0.080 | 0.864 |
| Intercept (β_0_) | | -4.58 | 0.074 | 0.00 |
| LL (β_1_) | | 0.10 | 0.001 | 0.00 |
| LW(β_2_) | | 0.35 | 0.004 | 0.00 |

*Multiple linear regression: LA=β_0_+β_1_LL+β_2_LW+∑_g_γ_g_D_g_, with Gemlik as the reference (all dummies 0). The coef reports γ_g_​ (intercept shift vs. Gemlik), std_err its standard error, and *p*value the two-sided p-value. Positive (negative) coefficients indicate higher (lower) mean LA than Gemlik at fixed LL and LW.
