# Supplementary material for: Artificial intelligence-based modeling for accurate leaf area estimation in olive (Olea europaea L.) cultivars
Source: PLoS One. 2026 Jan 2;21(1):e0339865. doi: 10.1371/journal.pone.0339865 (PMC12758791; doi:10.1371/journal.pone.0339865)
Supplement: S3 Table — (DOCX) [file pone.0339865.s003.docx]

**S3 Table.** Paired-sample t-test results for comparison of LA estimation methods.

| Cultivars | | SD of difference | P value | 95% Confidence interval of the difference | |
| --- | --- | --- | --- | --- | --- |
|  |  |  |  | Lower | Upper |
| Arbequina' | Observed and MLR model | 0.24 | 0.79 | -0.07 | 0.09 |
|  | Observed and ANN model | 0.19 | 0.10 | -0.01 | 0.11 |
|  | MLR and ANN | 0.24 | 0.30 | -0.04 | 0.12 |
| Ayvalık' | Observed and MLR model | 0.30 | 0.00 | -0.25 | -0.06 |
|  | Observed and ANN model | 0.24 | 0.99 | -0.08 | 0.08 |
|  | MLR and ANN | 0.25 | 0.00 | 0.08 | 0.24 |
| Çelebi' | Observed and MLR model | 0.26 | 0.97 | -0.08 | 0.09 |
|  | Observed and ANN model | 0.30 | 0.00 | -0.44 | -0.25 |
|  | MLR and ANN | 0.31 | 0.00 | -0.45 | -0.25 |
| Domat' | Observed and MLR model | 0.57 | 0.38 | -0.10 | 0.26 |
|  | Observed and ANN model | 0.68 | 0.00 | 0.82 | 1.24 |
|  | MLR and ANN | 0.67 | 0.00 | 0.75 | 1.16 |
| Edincik Su' | Observed and MLR model | 0.31 | 0.80 | -0.08 | 0.11 |
|  | Observed and ANN model | 0.42 | 0.00 | 0.32 | 0.58 |
|  | MLR and ANN | 0.44 | 0.00 | 0.30 | 0.58 |
| Elmacık' | Observed and MLR model | 0.14 | 0.13 | -0.01 | 0.08 |
|  | Observed and ANN model | 0.14 | 0.00 | 0.08 | 0.16 |
|  | MLR and ANN | 0.12 | 0.00 | 0.05 | 0.13 |
| Frantoio' | Observed and MLR model | 0.46 | 0.99 | -0.14 | 0.14 |
|  | Observed and ANN model | 0.39 | 0.00 | -0.33 | -0.09 |
|  | MLR and ANN | 0.28 | 0.00 | -0.30 | -0.12 |
| Gemlik' | Observed and MLR model | 0.22 | 0.45 | -0.04 | 0.10 |
|  | Observed and ANN model | 0.20 | 0.06 | -0.12 | 0.00 |
|  | MLR and ANN | 0.23 | 0.02 | -0.16 | -0.01 |
| Gemlik-21' | Observed and MLR model | 0.32 | 0.99 | -0.10 | 0.10 |
|  | Observed and ANN model | 0.23 | 0.33 | -0.04 | 0.11 |
|  | MLR and ANN | 0.27 | 0.39 | -0.05 | 0.12 |
| Girit Zeytini' | Observed and MLR model | 0.13 | 0.27 | -0.06 | 0.02 |
|  | Observed and ANN model | 0.09 | 0.00 | -0.11 | -0.06 |
|  | MLR and ANN | 0.10 | 0.00 | -0.09 | -0.03 |
| Halhalı' | Observed and MLR model | 0.23 | 0.94 | -0.07 | 0.07 |
|  | Observed and ANN model | 0.15 | 0.01 | 0.01 | 0.11 |
|  | MLR and ANN | 0.16 | 0.02 | 0.01 | 0.11 |
| Karamani' | Observed and MLR model | 0.25 | 0.42 | -0.11 | 0.05 |
|  | Observed and ANN model | 0.20 | 0.00 | -0.29 | -0.17 |
|  | MLR and ANN | 0.30 | 0.00 | -0.29 | -0.11 |
| Kilis Yağlık' | Observed and MLR model | 0.35 | 0.46 | -0.07 | 0.15 |
|  | Observed and ANN model | 0.38 | 0.00 | -0.45 | -0.21 |
|  | MLR and ANN | 0.35 | 0.00 | -0.48 | -0.26 |
| Manzanilla' | Observed and MLR model | 0.25 | 0.40 | -0.05 | 0.11 |
|  | Observed and ANN model | 0.19 | 0.00 | -0.24 | -0.12 |
|  | MLR and ANN | 0.18 | 0.00 | -0.27 | -0.16 |
| Memecik' | Observed and MLR model | 0.25 | 0.59 | -0.06 | 0.10 |
|  | Observed and ANN model | 0.23 | 0.40 | -0.10 | 0.04 |
|  | MLR and ANN | 0.19 | 0.09 | -0.11 | 0.01 |
| Nizip Yağlık' | Observed and MLR model | 0.57 | 0.76 | -0.15 | 0.21 |
|  | Observed and ANN model | 0.64 | 0.00 | -0.53 | -0.13 |
|  | MLR and ANN | 0.41 | 0.00 | -0.48 | -0.23 |
| Sarı Haşebi' | Observed and MLR model | 0.31 | 0.78 | -0.08 | 0.11 |
|  | Observed and ANN model | 0.19 | 0.00 | 0.08 | 0.20 |
|  | MLR and ANN | 0.25 | 0.00 | 0.04 | 0.20 |
| Sarı Ulak' | Observed and MLR model | 0.32 | 0.96 | -0.10 | 0.10 |
|  | Observed and ANN model | 0.20 | 0.00 | -0.31 | -0.19 |
|  | MLR and ANN | 0.25 | 0.00 | -0.33 | -0.17 |
| Sarı Yaprak' | Observed and MLR model | 0.41 | 0.50 | -0.17 | 0.08 |
|  | Observed and ANN model | 0.30 | 0.81 | -0.08 | 0.11 |
|  | MLR and ANN | 0.26 | 0.19 | -0.03 | 0.14 |
| Saurani' | Observed and MLR model | 0.19 | 0.23 | -0.02 | 0.09 |
|  | Observed and ANN model | 0.12 | 0.72 | -0.03 | 0.04 |
|  | MLR and ANN | 0.16 | 0.25 | -0.08 | 0.02 |
| Tavşan Yüreği' | Observed and MLR model | 0.43 | 0.36 | -0.19 | 0.07 |
|  | Observed and ANN model | 0.36 | 0.01 | 0.05 | 0.28 |
|  | MLR and ANN | 0.31 | 0.00 | 0.13 | 0.32 |
| Uslu' | Observed and MLR model | 0.34 | 0.80 | -0.12 | 0.09 |
|  | Observed and ANN model | 0.32 | 0.81 | -0.09 | 0.11 |
|  | MLR and ANN | 0.30 | 0.58 | -0.07 | 0.12 |
| All | Observed and MLR model | 0.43 | 1.00 | -0.01 | 0.03 |
|  | Observed and ANN model | 0.56 | 0.74 | -0.04 | 0.03 |
|  | MLR and ANN | 0.37 | 0.61 | -0.02 | 0.03 |
