# Supplementary material for: Artificial intelligence-based modeling for accurate leaf area estimation in olive (Olea europaea L.) cultivars
Source: PLoS One. 2026 Jan 2;21(1):e0339865. doi: 10.1371/journal.pone.0339865 (PMC12758791; doi:10.1371/journal.pone.0339865)
Supplement: S4 Table — (DOCX) [file pone.0339865.s004.docx]

**S4 Table.** Hyper-parameter and functions selected during the training phase.

| **Model Parameters and functions** | **(ANN)2-3-1** | **(ANN)3-4-1** |
| --- | --- | --- |
| Number of hidden layers | 1 | 1 |
| Number of hidden neurons | 3 | 4 |
| Learning Rate | 0.01 | 0.01 |
| Algorithm | Levenberg–Marquardt | Levenberg–Marquardt |
| Activation function in hidden Layer | Tansig | Tansig |
| Activation function in output Layer | Purelin | Purelin |
| Number of epochs | 100-500 (100 iterations) | 100-500 (100 iterations) |
| Network structure | 2-3-1 | 3-4-1 |
