# Supplementary material for: Artificial intelligence-based modeling for accurate leaf area estimation in olive (Olea europaea L.) cultivars
Source: PLoS One. 2026 Jan 2;21(1):e0339865. doi: 10.1371/journal.pone.0339865 (PMC12758791; doi:10.1371/journal.pone.0339865)
Supplement: S5 Table — (DOCX) [file pone.0339865.s005.docx]

**S5 Table.** Multiple linear regression leaf area models were developed for olive cultivar.

| Cultivars | Model | Train | | | | Test | | | |
| --- | --- | --- | --- | --- | --- | --- | --- | --- | --- |
|  |  | R^2^ | MBE | MAE | RMSE | R^2^ | MBE | MAE | RMSE |
| ‘Arbequina’ | LA=-4.23+0.093LL+0.352LW | 0.99 | 0.01 | 0.21 | 0.24 | 0.98 | 0.43 | 0.45 | 0.52 |
| ‘Ayvalık’ | LA=-5.11+0.11LL+0.365LW | 0.99 | -0.16 | 0.27 | 0.34 | 0.98 | 0.13 | 0.23 | 0.31 |
| ‘Çelebi’ | LA=-4.987+0.102LL+0.35LW | 0.98 | 0.00 | 0.21 | 0.26 | 0.99 | -0.03 | 0.17 | 0.20 |
| ‘Domat’ | LA=-6.32+0.138LL+0.42LW | 0.97 | 0.08 | 0.48 | 0.58 | 0.97 | 0.77 | 0.78 | 0.90 |
| ‘Edincik Su’ | LA=-4.89+0.105LL+0.405LW | 0.99 | 0.01 | 0.26 | 0.31 | 0.96 | -0.11 | 0.24 | 0.29 |
| ‘Elmacık’ | LA=-2.245+0.08LL+0.216LW | 0.97 | 0.03 | 0.07 | 0.10 | 0.99 | -0.65 | 1.52 | 0.77 |
| ‘Frantoio’ | LA=-7.14+0.114LL+0.45LW | 0.97 | 0.01 | 0.35 | 0.46 | 0.98 | 0.21 | 0.35 | 0.45 |
| ‘Gemlik’ | LA=-4.24+0.104LL+0.305LW | 0.99 | 0.03 | 0.18 | 0.23 | 0.97 | 0.13 | 0.20 | 0.28 |
| ‘Gemlik-21’ | LA=-4.17+0.084LL+0.395LW | 0.98 | 0.00 | 0.27 | 0.32 | 0.97 | -0.14 | 0.22 | 0.26 |
| ‘Girit Zeytini’ | LA=-2.33+0.082LL+0.2076LW | 0.97 | -0.02 | 0.11 | 0.13 | 0.99 | 0.05 | 0.14 | 0.21 |
| ‘Halhalı’ | LA=-4.57+0.096LL+0.36LW | 0.98 | 0.00 | 0.19 | 0.23 | 0.96 | 0.62 | 0.64 | 0.71 |
| ‘Karamani’ | LA=-4.25+0.09LL+0.34LW | 0.99 | -0.03 | 0.22 | 0.25 | 0.97 | 0.28 | 0.34 | 0.40 |
| ‘Kilis Yağlık’ | LA=-4.83+0.103LL+0.34LW | 0.98 | 0.04 | 0.29 | 0.36 | 0.96 | 0.32 | 0.34 | 0.44 |
| ‘Manzanilla’ | LA=-5.7+0.113LL+0.365LW | 0.99 | 0.03 | 0.20 | 0.26 | 0.98 | 0.45 | 0.45 | 0.57 |
| ‘Memecik’ | LA=-4.05+0.106LL+0.28LW | 0.98 | 0.02 | 0.20 | 0.26 | 0.98 | 0.18 | 0.23 | 0.27 |
| ‘Nizip Yağlık’ | LA=-4.5+0.11LL+0.289LW | 0.91 | 0.03 | 0.36 | 0.58 | 0.98 | 0.27 | 0.33 | 0.38 |
| ‘Sarı Haşebi’ | LA=-4.73+0.077LL+0.478LW | 0.98 | 0.01 | 0.24 | 0.32 | 0.92 | 0.07 | 0.11 | 0.14 |
| ‘Sarı Ulak’ | LA=-5.895+0.114LL+0.37LW | 0.97 | 0.00 | 0.26 | 0.32 | 0.98 | 0.49 | 0.60 | 0.79 |
| ‘Sarı Yaprak’ | LA=-7.46+0.127LL+0.44LW | 0.97 | -0.05 | 0.35 | 0.42 | 0.98 | 1.14 | 1.14 | 1.32 |
| ‘Saurani’ | LA=-2.91+0.081LL+0.273LW | 0.99 | 0.01 | 0.08 | 0.12 | 0.92 | 0.19 | 0.23 | 0.25 |
| ‘Tavşan Yüreği’ | LA=-6.16+0.13LL+0.36LW | 0.98 | -0.07 | 0.38 | 0.44 | 0.98 | 0.31 | 0.53 | 0.68 |
| ‘Uslu’ | LA=-4.64+0.084LL+0.42LW | 0.98 | -0.01 | 0.29 | 0.35 | 0.98 | -0.03 | 0.27 | 0.36 |

*LA* leaf area (cm^2^), *LW* leaf width (cm), *LL* leaf length (cm), *R^2^* coefficient of determination, *MBE* mean bias error, *MAE* mean absolute error, *RMSE* root mean square error
