# Supplementary material for: Artificial intelligence-based modeling for accurate leaf area estimation in olive (Olea europaea L.) cultivars
Source: PLoS One. 2026 Jan 2;21(1):e0339865. doi: 10.1371/journal.pone.0339865 (PMC12758791; doi:10.1371/journal.pone.0339865)
Supplement: S6 Table — (DOCX) [file pone.0339865.s006.docx]

**S6 Table.** Weight values between the input and hidden layers of the ANN model incorporating dummy (one-hot) encoded cultivar variables (reference: ‘Gemlik’).

| Dummy | W_1i_ | W_2i_ | W_3i_ | W_4i_ |
| --- | --- | --- | --- | --- |
| D_Arbequina | 0.176 | -0.001 | 0.524 | -0.069 |
| D_Ayvalık | 0.269 | 0.196 | 0.349 | 0.012 |
| D_Çelebi | 0.006 | 0.015 | 0.017 | 0.022 |
| D_Domat | 0.030 | 0.497 | -0.189 | -0.113 |
| D_Edincik Su | 0.254 | -0.013 | 0.261 | 0.004 |
| D_Elmacık | 0.105 | -0.034 | 0.513 | -0.113 |
| D_Frantoio | -0.276 | -0.174 | 0.092 | -0.073 |
| D_Gemlik-21 | -0.030 | -0.117 | 0.015 | -0.016 |
| D_Girit Zeytini | 0.253 | 0.077 | 0.215 | 0.181 |
| D_Halhalı | 0.217 | -0.028 | 0.442 | -0.020 |
| D_Karamani | -0.379 | -0.234 | 0.101 | -0.075 |
| D_Kilis Yağlık | -0.001 | -0.058 | 0.018 | 0.023 |
| D_Manzanilla | -0.285 | -0.164 | 0.087 | -0.078 |
| D_Memecik | -0.712 | -0.135 | 0.090 | -0.089 |
| D_Nizip Yağlık | 0.321 | 0.467 | 0.697 | -0.074 |
| D_Sarı Haşebi | -0.053 | -0.136 | 0.015 | -0.042 |
| D_Sarı Ulak | 0.358 | 0.573 | 0.400 | -0.045 |
| D_Sarı Yaprak | -0.060 | 0.057 | -0.011 | -0.036 |
| D_Saurani | 0.314 | 0.339 | 0.224 | 0.015 |
| D_Tavşan Yüreği | 0.176 | -0.030 | 0.621 | -0.071 |
| D_Uslu | -0.091 | -0.146 | 0.039 | -0.041 |
| LL | 0.288 | -0.706 | -0.506 | -0.264 |
| LW | 0.534 | 0.708 | -0.583 | -0.207 |
| Bias 1 | -0.486 | -1.005 | 0.085 | 1.083 |

The Supplementary Table S4 lists the connection weights (*W₁ᵢ–W₄ᵢ*) and bias terms from the input layer (LL, LW, and cultivar dummies) to the hidden layer neurons in the 3-4-1 network architecture. Positive and negative weights indicate the direction and magnitude of each variable’s contribution to the hidden layer activation.
