# Supplementary material for: Artificial intelligence-based modeling for accurate leaf area estimation in olive (Olea europaea L.) cultivars
Source: PLoS One. 2026 Jan 2;21(1):e0339865. doi: 10.1371/journal.pone.0339865 (PMC12758791; doi:10.1371/journal.pone.0339865)
Supplement: S1 Note — (DOCX) [file pone.0339865.s007.docx]

**S1 Note.** Normalization of input and output data

All input and output variables were normalized in the range of 0–1 to meet the requirements of the machine learning models before the training and testing phases using Eq. (S1).

| X_norm_=$\frac{Xa-Xmin}{Xmax-Xmin}$ | (S1) |
| --- | --- |

where *X*_norm_ is the normalized value of a variable; *X*_a_ is the measured value of a variable and; *X*_max_ and *X*_min_ are the measured maximum and minimum values of a variable.
