# Supplementary material for: Artificial intelligence-based modeling for accurate leaf area estimation in olive (Olea europaea L.) cultivars
Source: PLoS One. 2026 Jan 2;21(1):e0339865. doi: 10.1371/journal.pone.0339865 (PMC12758791; doi:10.1371/journal.pone.0339865)
Supplement: S2 Note — (DOCX) [file pone.0339865.s008.docx]

**S2 Note.** Mathematical formulation of the ANN model

The input vector was defined as *X = [LL, LW, D]*, where *LL* and *LW* denote leaf length and width, and *D* is the one-hot encoded cultivar vector (21 dummy variables, reference: ‘Gemlik’).

Weight matrices and bias vectors were denoted as *W₁ ∈ ℝ⁴×²³*, *b₁ ∈ ℝ⁴*, *W₂ ∈ ℝ¹×⁴*, and *b₂ ∈ ℝ*. The forward pass was expressed as:

*u = W₁X + b₁*
*h = tanh(u)*
*LA = W₂h + b₂*

The scalar form and compact expression used for network computation were:

*u_j = b₁ⱼ + Σₖ wⱼₖ⁽¹⁾ xₖ*
*hⱼ = tanh(uⱼ) = 2/(1 + e^(−2uⱼ)) − 1,* j = 1,…,4
*LA = b₂ + Σⱼ w₁ⱼ⁽²⁾ hⱼ*

*LA = W₂ tanh(W₁ [LL, LW, D] + b₁) + b₂*
