# Supplementary figures and images for: Artificial intelligence-based modeling for accurate leaf area estimation in olive (Olea europaea L.) cultivars
Source: PLoS One. 2026 Jan 2;21(1):e0339865. doi: 10.1371/journal.pone.0339865 (PMC12758791; doi:10.1371/journal.pone.0339865)

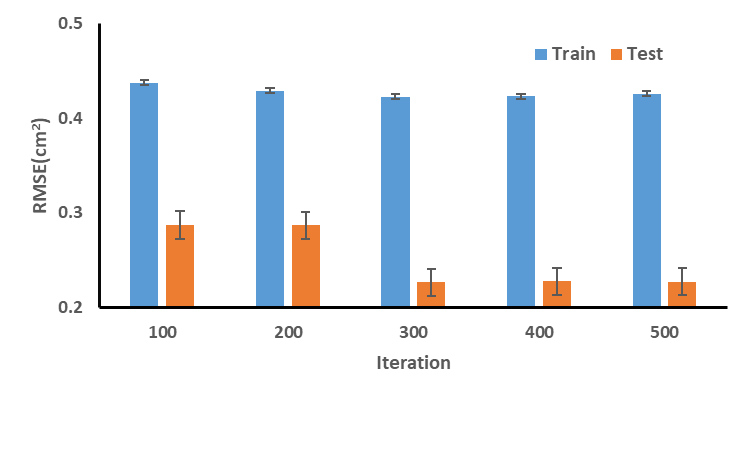


**S1 Fig.** Iteration RMSE change according to 2-3-1 network structure.

Supplement: S1 Fig — (DOCX) [file pone.0339865.s009.docx]

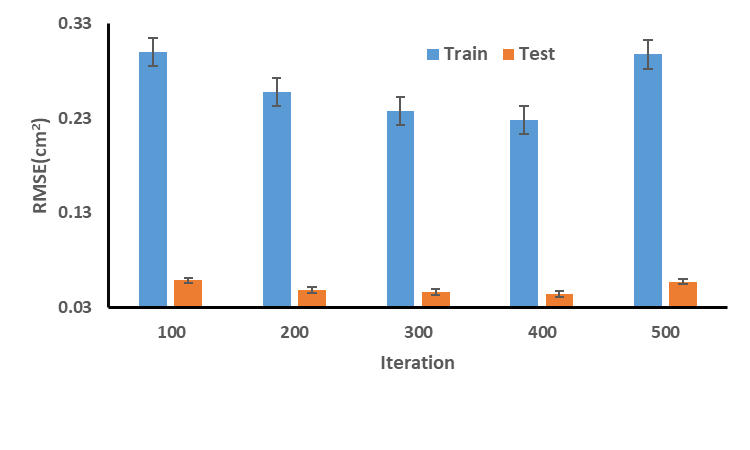


**S2 Fig.** Iteration RMSE change according to 3-4-1 network structure.

Supplement: S2 Fig — (DOCX) [file pone.0339865.s010.docx]
