# Supplementary material for: Artificial intelligence-based modeling for accurate leaf area estimation in olive (Olea europaea L.) cultivars
Source: PLoS One. 2026 Jan 2;21(1):e0339865. doi: 10.1371/journal.pone.0339865 (PMC12758791; doi:10.1371/journal.pone.0339865)
Supplement: S1 Code — (DOCX) [file pone.0339865.s011.docx]

**S1 Code.** The MATLAB codes used.

%% ===========================================================

% 5-Fold MLP (tansig -> purelin, trainlm), 100:100:500 iterations

% Data: Excel, 3 columns (X1, X2 = inputs; Y = output). First row = header.

% Outputs: Metrics, weights/biases, indexes, data and predictions,

% summary tables -> multi-sheet Excel file.

% Note: At each iteration, the same initial weights are restored (fair comparison).

% ============================================================

clear; clc;

%% === USER SETTINGS ======================================================

excelGirisDosya = 'veri.xlsx'; % <-- 3-column data file (first row = header)

Excel output = 'MLP_5Fold_LM_Sonuclar.xlsx';

hiddenSize = 10; % number of hidden neurons (change if desired)

iterList = 100:100:500; % desired iterations

numFolds = 5; % 5 "folds", each with 70% training / 30% testing (HoldOut)

rng(42); % fix for reproducibility

%% === READ DATA ==========================================================

T = readtable(excelGirisDosya); % assumes first row as header

if width(T) < 3

error('Excel sheet must have at least 3 columns (X1, X2, Y).');

end

% First two columns: X (inputs), 3rd column: Y (output).

X = T{:,1:2};

Y = T{:,3};

% Handle missing values

nanMask = any(isnan([X Y]),2);

if any(nanMask)

warning('%d rows contain NaN and were removed.', sum(nanMask));

X(nanMask,:) = [];

Y(nanMask,:) = [];

end

N = size(X,1);

if N < 10

error('Data appears too small (N<10).');

end

%% === SUMMARY TABLE PREPARATION ==========================================

% Columns: Fold, Iter, Split, MBE, MAE, RMSE, R2

summaryRows = {};

summaryHeader = {'Fold','Iter','Split','MBE','MAE','RMSE','R2'};

% Main process: 5 independent HoldOut folds

for f = 1:numFolds

% --- 70% training / 30% testing split ---

c = cvpartition(N,'HoldOut',0.30);

trIdx = training(c); % logical index

teIdx = test(c);

Xtr = X(trIdx,:); Ytr = Y(trIdx,:);

Xte = X(teIdx,:); Yte = Y(teIdx,:);

% Fold-specific sheet: indexes

foldSplitSheet = sprintf('F%d_Split', f);

writetable(table(find(trIdx), 'VariableNames', {'Train_RowIndex'}), ...

Excel output, 'Sheet', foldSplitSheet, 'WriteMode','overwritesheet');

writetable(table(find(teIdx), 'VariableNames', {'Test_RowIndex'}), ...

Excel output, 'Sheet', foldSplitSheet, 'WriteMode','append');

% Create initial network: will be saved to restore same start point each iteration

% (configure + init once, save weights as wb0)

net0 = feedforwardnet(hiddenSize, 'trainlm');

net0.layers{1}.transferFcn = 'tansig';

net0.layers{2}.transferFcn = 'purelin';

net0.trainParam.showWindow = false; % disable GUI

net0.trainParam.max_fail = 6; % (no val set but keep default)

net0.divideFcn = 'divideind'; % we will provide indices

% No validation set

net0.divideParam.trainInd = 1:size(Xtr,1);

net0.divideParam.valInd = [];

net0.divideParam.testInd = [];

% Configure inputs/outputs as column vectors

net0 = configure(net0, Xtr', Ytr');

net0 = init(net0); % random initialization

wb0 = getwb(net0); % initial weights + biases packed

% Save raw training and test sets (with variable names)

baseDataSheet = sprintf('F%d_Data', f);

writetable(array2table([Xtr Ytr], ...

'VariableNames', [T.Properties.VariableNames(1:2), T.Properties.VariableNames(3)]), ...

Excel output, 'Sheet', baseDataSheet, 'WriteMode','overwritesheet');

writetable(array2table([Xte Yte], ...

'VariableNames', [T.Properties.VariableNames(1:2), T.Properties.VariableNames(3)]), ...

Excel output, 'Sheet', baseDataSheet, 'WriteMode','append');

% Iteration loop

for it = iterList

% Copy network and restore initial weights

net = net0;

net = setwb(net, wb0);

net.trainParam.epochs = it;

% Split: training/test (no val)

net.divideFcn = 'divideind';

net.divideParam.trainInd = 1:size(Xtr,1);

net.divideParam.valInd = [];

net.divideParam.testInd = [];

% Train

[net, tr] = train(net, Xtr', Ytr');

% Predictions

yhat_tr = net(Xtr')'; % training

yhat_te = net(Xte')'; % testing

% Metrics

[mbe_tr, mae_tr, rmse_tr, r2_tr] = metrics(Ytr, yhat_tr);

[mbe_te, mae_te, rmse_te, r2_te] = metrics(Yte, yhat_te);

% ---- Summary metric rows (train/test) ----

summaryRows(end+1,:) = {f, it, 'Train', mbe_tr, mae_tr, rmse_tr, r2_tr}; %#ok<SAGROW>

summaryRows(end+1,:) = {f, it, 'Test', mbe_te, mae_te, rmse_te, r2_te}; %#ok<SAGROW>

% ---- Weights and biases ----

IW11 = net.IW{1,1}; % [hiddenSize x 2]

b1 = net.b{1}; % [hiddenSize x 1]

LW21 = net.LW{2,1}; % [1 x hiddenSize]

b2 = net.b{2}; % [1 x 1]

wbSheet = sprintf('F%d_I%d_Weights', f, it);

writematrix(IW11, Excel output, 'Sheet', wbSheet, 'Range','A1');

writematrix(b1, Excel output, 'Sheet', wbSheet, 'Range','A1', 'WriteMode','append');

% Add headers:

Wtab = array2table(IW11);

Wtab.Properties.VariableNames = compose('W1_%d', 1:size(IW11,2));

Wtab.Properties.RowNames = compose('H%02d', 1:size(IW11,1));

writetable(resetRowNames(Wtab), Excel output, 'Sheet', wbSheet, 'Range','A1', 'WriteMode','overwritesheet');

B1tab = table(b1, 'VariableNames', {'b1'});

writetable(B1tab, Excel output, 'Sheet', wbSheet, 'Range','A2', 'WriteMode','append');

LWtab = array2table(LW21, 'VariableNames', compose('H%02d', 1:size(LW21,2)));

writetable(LWtab, Excel output, 'Sheet', wbSheet, 'Range','C1', 'WriteMode','append');

B2tab = table(b2, 'VariableNames', {'b2'});

writetable(B2tab, Excel output, 'Sheet', wbSheet, 'Range','C3', 'WriteMode','append');

% ---- Metric table (this iteration) ----

metSheet = sprintf('F%d_I%d_Metrics', f, it);

Met = table( ...

["Train"; "Test"], ...

[mbe_tr; mbe_te], [mae_tr; mae_te], [rmse_tr; rmse_te], [r2_tr; r2_te], ...

'VariableNames', {'Split','MBE','MAE','RMSE','R2'});

writetable(Met, Excel output, 'Sheet', metSheet, 'WriteMode','overwritesheet');

% ---- Training/Test data & predictions tables (this iteration) ----

trainTab = table(Xtr(:,1), Xtr(:,2), Ytr, yhat_tr, ...

'VariableNames', {'X1','X2','Y_true','Y_pred'});

testTab = table(Xte(:,1), Xte(:,2), Yte, yhat_te, ...

'VariableNames', {'X1','X2','Y_true','Y_pred'});

writetable(trainTab, Excel output, 'Sheet', sprintf('F%d_I%d_DataTrain', f, it), 'WriteMode','overwritesheet');

writetable(testTab, Excel output, 'Sheet', sprintf('F%d_I%d_DataTest', f, it), 'WriteMode','overwritesheet');

% ---- Training process info (iteration count, performance, etc.) ----

% write some summaries from tr struct:

trInfo = table( ...

it, tr.numepochs, tr.best_epoch, ...

'VariableNames', {'TargetEpochs','NumEpochs','BestEpoch'});

writetable(trInfo, Excel output, 'Sheet', sprintf('F%d_I%d_TrainInfo', f, it), 'WriteMode','overwritesheet');

end % iteration

end % fold

%% === WRITE SUMMARY TABLES ===============================================

Summary = cell2table(summaryRows, 'VariableNames', summaryHeader);

writetable(Summary, Excel output, 'Sheet', 'Summary_All', 'WriteMode', 'overwritesheet');

% Fold averages (by iteration and split)

G = groupsummary(Summary, {'Iter','Split'}, 'mean', {'MBE','MAE','RMSE','R2'});

writetable(G, Excel output, 'Sheet', 'Summary_MeanByIter', 'WriteMode','overwritesheet');

disp('Done. Results written to MLP_5Fold_LM_Sonuclar.xlsx.');

%% === HELPER FUNCTIONS ===================================================

function [mbe, mae, rmse, r2] = metrics(y_true, y_pred)

% MBE: mean(pred - true)

e = y_pred - y_true;

mbe = mean(e);

mae = mean(abs(e));

rmse= sqrt(mean(e.^2));

ss_res = sum((y_true - y_pred).^2);

ss_tot = sum((y_true - mean(y_true)).^2);

r2 = 1 - ss_res/ss_tot;

end

function T2 = resetRowNames(T1)

% since writetable does not support row names, convert them to a normal column

if ~isempty(T1.Properties.RowNames)

T2 = addvars(T1, string(T1.Properties.RowNames), 'Before', 1, 'NewVariableNames', "Row");

T2.Properties.RowNames = {};

else

T2 = T1;

end

end
