# Supplementary material for: Artificial intelligence-based modeling for accurate leaf area estimation in olive (Olea europaea L.) cultivars
Source: PLoS One. 2026 Jan 2;21(1):e0339865. doi: 10.1371/journal.pone.0339865 (PMC12758791; doi:10.1371/journal.pone.0339865)
Supplement: Attachment — Submitted filename: Revie report.docx [file pone.0339865.s012.docx]

**Reviewer Report**

**Manuscript Title:** Artificial intelligence-based modeling for accurate leaf area estimation in olive (*Olea europaea* L.) cultivars

**Manuscript ID:** PONE-D-25-55767

**Journal:** PLOS ONE

**General Assessment**

The manuscript presents a comprehensive study comparing Multiple Linear Regression (MLR) and Artificial Neural Network (ANN) models for leaf area estimation in 22 olive cultivars. The topic is relevant to precision agriculture and phenotyping research, offering practical tools for non-destructive plant measurements. The work is generally well-structured, methodologically sound, and supported with appropriate statistical analysis. However, several aspects require clarification, tightening, and improvement to enhance scientific rigor and reproducibility.

**Strengths**

1. **Novelty and relevance:** The study fills a practical gap in olive phenotyping by developing cultivar-specific and general predictive models using easily measurable traits (leaf length and width).
2. **Methodological rigor:** Both MLR and ANN approaches were implemented and validated using adequate datasets (n = 1320) and appropriate cross-validation.
3. **Comprehensive comparison:** The performance metrics (R², RMSE, MAE, MBE) are clearly presented and allow a fair comparison between modeling approaches.
4. **High predictive accuracy:** ANN performance (R² up to 0.995) demonstrates strong potential for agricultural applications.
5. **Practical implications:** The models could be useful in high-throughput phenotyping and field-scale precision agriculture systems.

**Major Comments**

1. **Model reproducibility and data sharing**
   - Although the authors claim that “all relevant data are within the manuscript,” the training/testing datasets and MATLAB codes are not provided. For transparency and reproducibility, numerical datasets or example code should be made available in supplementary materials or a public repository (e.g., Zenodo or GitHub).
2. **Statistical validation**
   - The manuscript would benefit from statistical comparison of ANN vs. MLR performance (e.g., paired t-test or Wilcoxon test on residual errors) to confirm whether ANN’s superiority is statistically significant.
   - Overfitting assessment for ANN is limited. Please report training vs. validation loss curves or early stopping criteria.
3. **Model architecture details**
   - While the ANN topology is described (2–3–1 and 3–4–1 structures), key hyperparameters such as learning rate, epoch number, and data normalization procedures are missing. These are necessary for reproducibility.
4. **Cultivar-specific effects**
   - The manuscript reports limited cultivar effects after multiple testing correction. However, a deeper biological interpretation of why certain cultivars (e.g., Domat, Elmacık) deviate would improve discussion quality.
5. **Figures and Tables**
   - Figures 4 and 5 need clearer axes labels and consistent units. Include regression equations and R² values directly on the plots for readability.
   - Table 2 is extensive but could be moved to supplementary information; summary tables (mean R², RMSE, etc.) should be included in the main text.
6. **Language and structure**
   - The manuscript is generally clear but verbose in several sections (especially Introduction and Discussion). Condensing redundant literature descriptions would improve readability.
   - Ensure all scientific names (e.g., *Olea europaea L.*) are italicized.

**Minor Comments**

1. Check consistency of abbreviations (e.g., LL, LW, LA) throughout the text and figures.
2. Include units in all table headers (e.g., mm, cm²).
3. Provide references for software tools (MATLAB R2010b, Excel 2015) in Methods.
4. Correct minor typographical errors (e.g., extra spaces, inconsistent capitalization).
5. Ensure that the ethical and data availability statements comply fully with *PLOS ONE* requirements.

**Recommendation**

**Major Revision**

The study presents valuable and potentially publishable findings, but improvements in methodological transparency, statistical validation, and presentation are necessary before acceptance. Addressing the comments above will greatly strengthen the scientific rigor and impact of the manuscript.
